# Effects of different physical activity interventions on women's sleep: a systematic review and network meta-analysis

**DOI:** 10.3389/fpsyg.2025.1704980

**Published:** 2025-11-20

**Authors:** Shuang Li, Zixian Xiao, Hongyu Wang, Xiaolin Zhang, Kelei Guo, Ying Zhu, Jingtao Wu, Chenmu Li, Yuwen Shangguan, Junlai Zhou, Dong Li

**Affiliations:** 1 School of Physical Education and Health, Zhaoqing University, Zhaoqing, China; 2 School of Physical Education and Health, Guangxi Normal University, Guilin, China; 3 School of Physical Education, Leshan Normal University, Leshan, China; 4 Department of Sport and Exercise Sciences, Kunsan National University, Kunsan, Republic of Korea; 5 School of Physical Education, Hainan Normal University, Haikou, China

**Keywords:** women, quality of sleep, physical activity, network meta-analysis, sleep

## Abstract

**Background:**

Sleep disorders are common among women, affecting quality of life and increasing disease risks. Physical activity is a recognized method to improve sleep, but existing meta-analyses lack clear criteria for female groups, with confounding factors from those with diseases or poor sleep, and lack universal advice for the general female population. This study aims to compare and rank different physical activity intervention strategies to provide effective references for improving sleep quality in the general female population.

**Methods:**

A systematic search across four databases (PubMed, Cochrane Library, Embase, Web of Science) identified randomized controlled trials (RCTs) on physical activity for sleep in peri- and post-menopausal women. Search timeframe: database inception to June 2025. Two independent researchers selected studies, extracted data, and assessed quality via the Cochrane Risk of Bias Tool. We conducted frequentist network meta-analyses (Stata/SE 15.1) integrated direct and indirect evidence. Surface Under the Cumulative Ranking Curve (SUCRA) ranked interventions by efficacy probabilistically, with higher values indicating superior outcomes.

**Results:**

Fifteen RCTs were finally included in the meta—analysis for synthesis. The results showed that, in terms of the effect on sleep quality in women, aerobic exercise exhibited superior efficacy with a SUCRA value of 77.2%, followed by Multi mode motion with a SUCRA value of 70.5%. Mind body exercise and stretching exercise also showed improvements to a certain extent, with SUCRA values of 57.1% and 38.8%, respectively.

**Conclusion:**

Our findings confirm aerobic exercise appears most effective for improving women's sleep quality, followed by multimodal exercise. Future research should focus on exercise intensity and optimal pre-bedtime timing this will further optimize non-pharmacological interventions for female sleep disorders and provide an evidence base for community health management.

**Systematic review registration:**

https://www.crd.york.ac.uk/PROSPERO/view/CRD420251058655; identifier: CRD420251058655.

## 1 Introduction

Poor sleep quality affects a large portion of the population in daily life or with chronic diseases, and it is one of the most common problems in medical practice (Morin and Benca, 2012). It is mainly characterized by people's dissatisfaction with sleep duration or quality and difficulty falling asleep (Morin et al., 2015). If people continuously suffer from sleep disorders, it can easily lead to a decline in their quality of life and the development of physical and mental diseases (Roth and Roehrs, 2003). It may also impair cognitive ability, immune function, etc. (Halson, 2014). Poor sleep quality is usually associated with some factors, including modifiable ones like lifestyle habits and non-modifiable ones like gender (Uhlig et al., 2014). Gender is generally considered to play an important role, especially in women's sleep quality issues (Madrid-Valero et al., 2017). Women may experience poor sleep due to various factors (Bei et al., 2015), such as physical and mental states and hormone levels. The incidence of insomnia in women is about 1.41 times that in men (Zhang and Wing, 2006). Moreover, fluctuations in estrogen and progesterone levels in women's bodies can lead to a decline in sleep quality (Zeng et al., 2020; Krystal, 2003; Zhang et al., 2012; Blümel et al., 2012), making the impact of sleep quality issues more obvious. Sleep quality problems are common troubles that women are likely to encounter in their lives. They not only occur frequently during menopause (Hsu et al., 2005), but also have a significant impact on younger women (Fatima et al., 2016). When other diseases and emotional symptoms occur, these factors can also easily affect sleep quality through the side effects of relevant medications (Krystal, 2003). Therefore, women should pay attention to the issue of sleep quality.

To address the issue of sleep quality, people have proposed a series of measures. Among them, Pharmacological treatment is currently one of the most commonly prescribed treatment options for sleep problems. It mainly includes benzodiazepines and “non-benzodiazepines” (Donoghue and Lader, 2010). Despite dosage warnings, the use of these drugs remains relatively high, and they are the first choice for patients with sleep disorders (Lader, 2011). However, long term use of sleeping pills can have negative effects and even pose a risk of triggering other diseases. People who use benzodiazepines over the long term often become dependent on the drugs (Edinoff et al., 2021). Drug abuse can easily lead to a vicious cycle. However, research has found that women are more likely than men to be prescribed benzodiazepines (Olfson et al., 2015). Therefore, we can choose safer and more cost effective intervention measures (Krystal and Attarian, 2016). Considering the treatment cost and the comprehensive effectiveness on physical health, physical activity is an effective long term treatment method that helps prevent and treat insomnia (Amiri et al., 2021). Moreover, regular physical activity can improve subjective sleep quality (Xie et al., 2021). Compared with Pharmacological treatment and non-pharmacological intervention, physical activity can safely and effectively help patients with sleep disorders improve their sleep quality and enhance their quality of life by promoting overall health (Rios et al., 2019).

Physical activity is defined as any form of bodily movement caused by skeletal muscle contraction that results in energy expenditure (Caspersen et al., 1985), with the aim of preventing and treating diseases (Claes et al., 2017; Rosenbaum et al., 2014; Ströhle, 2009). Empirical studies indicate that different forms of physical activity can effectively enhance people's sleep quality (Bisson et al., 2019; Singh et al., 1997; Wang et al., 2020; Liu et al., 2023). In various diseases like cardiovascular diseases, diabetes, depression, and arthritis, physical activity is also regarded as an effective intervention for sleep disorders (Durcan et al., 2014; Løppenthin et al., 2014; Qiao et al., 2024). The physical activity can improve the sleep quality of different populations across their life cycles by regulating neurochemical levels, promoting cerebral blood flow, enhancing psychological resilience, and even directly improving sleep quality (Dolezal et al., 2017). Compared with Pharmacological treatment, physical activity has fewer side effects, higher cost effectiveness, and better patient compliance, which is especially crucial in the modern medical environment (Roine et al., 2009). Existing studies have analyzed the relationship between sleep quality and cardiopulmonary function (Dishman et al., 2015). It was found that as the physical fitness of female subjects declined and the duration of exercise decreased, the likelihood of women complaining about sleep increased. Researchers suggest that improving physical fitness may be an effective intervention for enhancing sleep quality. A similar study by Strand et al. also confirmed these findings, revealing that the control group with less engagement in physical activity faced a higher risk of insomnia (Chen et al., 2017). In a large-scale sample study, the correlation between exercise level and the incidence of insomnia symptoms further supported these viewpoints (Zheng et al., 2017). These studies all indicate that a lack of physical activity increases the risk of sleep disorders. Tailoring personalized exercise programs for women's sleep conditions, including appropriate types and intensities of physical activity, can further optimize treatment outcomes (Kline et al., 2013). These interventions can not only be used as standalone treatment methods but also be combined with Pharmacological treatment and psychotherapy to enhance clinical effectiveness (Monfaredi et al., 2022). Overall, interventions based on physical activity are a safe, effective, and easily implementable non-Pharmacological treatment strategy, especially suitable for patients seeking alternatives to drugs or concerned about their quality of life (Kredlow et al., 2015).

A recent meta-analysis showed that physical activity plays a positive role in improving women's sleep quality (Qiao et al., 2024). However, when recruiting participants, the authors failed to exclude those with other underlying diseases or comorbidities. This may have introduced significant confounding factors into the study population, thereby affecting the reliability of the results. Furthermore, current studies still have critical gaps in high-quality evaluations for selecting the optimal intervention among various physical activity methods. To bridge this gap and provide recommendations for precise intervention strategies for women's sleep quality, this study rigorously selected high-quality RCTs to conduct a systematic review and network meta-analysis. More importantly, compared with previous meta-analyses that included patient groups, this study takes “healthy adult women without chronic diseases or severe sleep disorders” as the core research object, and its additional value is reflected in three aspects: First, it eliminates the interference of confounding factors such as underlying diseases (e.g., diabetes, cardiovascular diseases) and drug side effects on intervention effects, making the causal link between “exercise type–sleep improvement” purer, and the results can more accurately reflect the real exercise benefits of healthy women; Second, it addresses the core need of healthy women to “prevent sleep problems rather than treat insomnia” and provides non-pharmacological sleep optimization solutions, filling the gap in existing studies that focus more on “treating patient populations” while neglecting “preventive care for healthy populations”; Third, through the quantitative ranking of network meta-analysis (instead of a single direct comparison), it clearly defines the priority ranking of exercise interventions for healthy women, avoiding blind trial-and-error when selecting sleep-enhancing exercises and making the recommendations more practical. Additionally, the female population was categorized into a more general conventional group. Comparative effectiveness evaluations and hierarchical rankings were conducted on different physical activity interventions to identify the optimal intervention method for improving women's sleep quality. This study provides more women with reference recommendations and exercise prescriptions for optimizing sleep quality.

## 2 Methods

### 2.1 Protocol and registration

We followed the requirements for literature inclusion, data organization, statistical analysis, and result reporting in the Preferred Reporting Items for Systematic Reviews and Meta-Analyses (PRISMA) 2020 guidelines. This study has also been registered in PROSPERO (CRD420251058655).

### 2.2 Selection criteria

The primary outcome measure in this study was sleep quality. In relevant RCTs, standardized tools were used to assess at least one sleep quality indicator, and data on the baseline and primary outcomes were provided. The main diagnostic tools are the Pittsburgh Sleep Quality Index (PSQI) and the Insomnia Severity Index (ISI). The PSQI is a standard tool for assessing sleep quality. As a self-rating questionnaire, the PSQI can effectively provide seven standardized indicators of overall sleep quality (Buysse et al., 1989). The ISI score is a reliable tool for quantifying perceived insomnia severity. It can be used as a screening tool or as an outcome measure in insomnia treatment research (Bastien et al., 2001).

### 2.3 Data sources and search strategy

We conducted a comprehensive search in four electronic database—PubMed, Cochrane Library, Embase, and Web of Science—for relevant literature on the relationship between physical activity and sleep quality in women. The search spanned from the establishment of each database to June 5, 2025. Following the PICOS (Population, Intervention, Comparison, Outcome, Study design) principle, the search terms included: “physical activity”, “physical activities”, “running”, “yoga”, “cycling”, “walking”, “tai chi”, “resistance training”, “pilates”, “qigong”, “acute exercise”, “women”, “female”, “Postmenopausal Women”, “Sleep Quality”, “insomnia”, “sleep disturbance”, “sleep”, “slumber quality”, “sleep disorder”, “Randomized controlled trial”, “controlled clinical trial”, “randomized”, “placebo”, “randomly”, “RCT”. For detailed search strategies, please consult Appendix B and B1.

### 2.4 Study selection

After conducting the literature search using the aforementioned search strategy, authors ZXX and SL independently performed the literature screening. The preliminary screening involved reviewing the titles and abstracts of the retrieved articles to identify potentially relevant studies. Subsequently, full-text reviews were conducted for the studies with higher relevance. Finally, studies that met the inclusion criteria were selected for statistical analysis. In case of any discrepancies, the team members engaged in discussions to reach a consensus.

### 2.5 Inclusion and exclusion criteria

The literature inclusion criteria were as follows:

1. The study subjects are clearly defined as females aged 18 or above.
2. The intervention measures include various forms of physical activity, such as aerobic exercise, Pilates, yoga, resistance training, and traditional Chinese fitness programs (e.g., qigong).
3. Standard sleep quality assessment indicators are used.
4. Only RCTs are included in the experiment.
5. The original data are provided.
6. The studies are published in full English text.

The literature exclusion criteria are as follows:

Studies that meet any of the following criteria will be excluded:

1. The study subjects are non-female individuals, pregnant women, and patients with diseases that may interfere with the assessment of sleep quality, such as severe sleep disorders, mental illnesses, other neurological diseases, diabetes, and cardiovascular diseases.
2. The intervention measures do not include physical activity.
3. The research types include qualitative studies, reviews, papers, and conference papers.
4. Non-interventional study designs include cross-sectional studies, case-control studies, and cohort studies.
5. The full text cannot be obtained or the experimental data is unavailable.
6. Non-English publications were excluded due to the research team's limited language proficiency, to avoid translation biases and interpretation errors and ensure accuracy.

### 2.6 Data extraction

The data extraction process was independently carried out by two researchers (SL and ZXX). Any discrepancies encountered during this process were resolved through group discussion. The following information was extracted from each study:

(1) Initial extraction: Data information was independently extracted by two researchers to ensure objective collection of information.
(2) Discrepancy resolution: Any discrepancies in the extracted data were resolved through group discussions until consensus was achieved.
(3) Information categorization: The following four categories of data were systematically extracted from each study:
   Basic study information: First author, publication year, and country/region where the study was conducted;
   Participant characteristics: Age, total sample size, and group allocation;
   Intervention details: Type of intervention, duration of intervention, weekly frequency, and total number of sessions;
   Outcome Measurement: Primary or secondary outcomes directly related to measuring sleep in women, as well as their corresponding measurement tools.
(4) Special data handling principles: For numerical information presented graphically but ambiguously described in text, Engauge Digitizer 12.1 software was used for digital extraction.

When a study reported multiple follow-up time points, preference was given to data assessed immediately after the intervention ended. In the absence of standard deviation (SD), SD values were estimated using the recommended formula from the Cochrane Handbook, utilizing the 95% confidence interval of the group means.

### 2.7 Quality assessment

We utilized the Cochrane Risk of Bias Assessment Tool (RoB2) to evaluate the quality of the studies based on five criteria: (1) the randomization process; (2) deviations from the intended interventions; (3) missing outcome data; (4) measurement of outcomes; and (5) selection of reported results. Based on this, we assessed the overall risk of bias for each study, classifying them as low risk, high risk, or having some concerns.

### 2.8 Statistical analysis

For continuous outcomes, we calculated the standardized mean difference (SMD) and its 95% confidence intervals (CIs). For statistical heterogeneity, we used the chi-square test *P*-value and I^2^ statistic (I^2^ > 50% = moderate heterogeneity; I^2^ > 75% = high heterogeneity). To resolve scale heterogeneity (e.g., varied measurement tools, intervention protocols), we applied an inverse variance-weighted random-effects model (accounting for between-study variability via τ^2^ estimation) to maintain methodological parsimony and comparative consistency. Per PRISMA-NMA guidelines, we prioritized a frequentist over Bayesian framework to improve interpretability and avoid Markov chain Monte Carlo convergence complexities. The analytical workflow included three core steps: (1) generating evidence network plots via Stata 15.1′s “network” package (node size = study sample size; line thickness = number of trials per comparison); (2) synthesizing effect sizes using maximum likelihood estimation in multivariate meta-regression (integrating direct/indirect evidence); (3) validating consistency via node-splitting tests (*p* > 0.05 = statistically consistent). For network meta-analysis, we used the “network” package to generate evidence network plots (as above). We calculated SUCRA (percentage values; higher = more effective) to rank intervention effectiveness, with results presented in a probability table. Potential publication bias was assessed via funnel plots, with adjustments for its potential impact on results.

## 3 Results

### 3.1 Trial selection

To ensure the accuracy of the literature search and screening process, two researchers (SL and ZXX) independently screened the titles, abstracts, and full texts after completing the literature search. The inter-rater reliability (Cohen's kappa) between these two researchers was calculated for both screening stages: the title and abstract screening phase and the full-text screening phase. The level of consistency was categorized as follows: moderate consistency (0.40–0.59), good consistency (0.60–0.74), and excellent consistency (>0.75).

**Figure 1:**
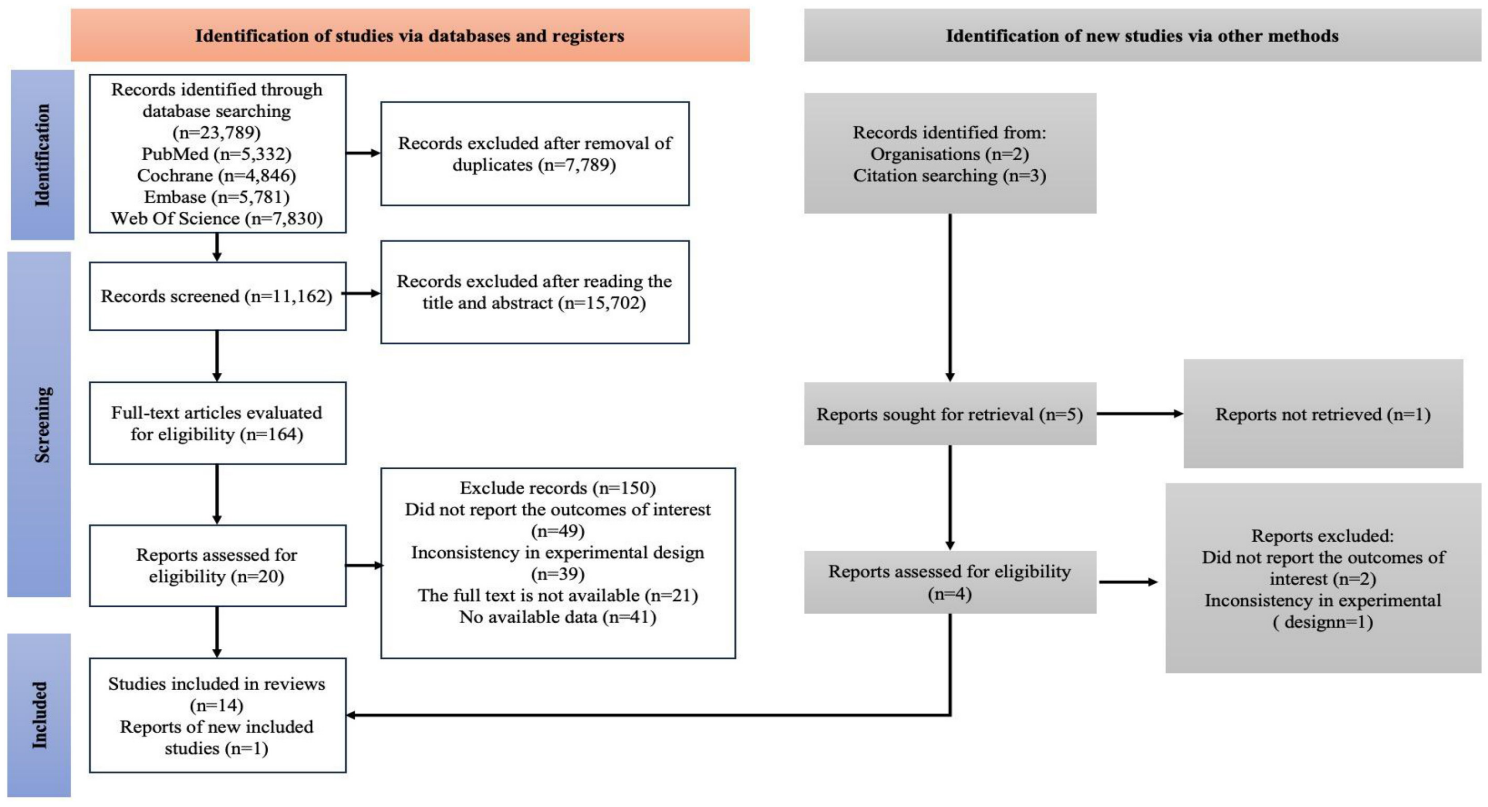
A summary of the evidence searches and selection process.

In the initial search, a comprehensive retrieval of four electronic databases was conducted from January 1, 2000, to June 5, 2025, and a total of 23,789 articles were identified. After removing duplicate studies (n = 7,789), 16,000 relevant articles remained. Subsequently, through screening of titles and abstracts, 15,702 articles were excluded, and finally, 164 articles advanced to the full-text review stage. At this stage, the scoring consistency between the two evaluators was rated as “good” (Cohen's kappa = 0.73). After the full text review, 149 articles were further excluded. Specifically, 49 articles did not report results, 39 had inconsistent experimental designs, 21 were unavailable in full text, and 40 had no available data. Therefore, a total of 15 studies were selected in the initial search (Figure 1). At this stage, the inter-rater reliability between the two assessors was classified as “excellent” (Cohen's kappa = 0.84).

### 3.2 Trial characteristics

Table 1 presents the characteristics of the included studies. All studies were published between the launch of the database and June 5, 2025. The sample sizes of the intervention groups in the included studies ranged from 10 to 103, with a total of 113 adult females. The sample sizes of the control groups ranged from 10 to 138, with a total of 148 females.

**Table 1:** Summary table of included reviews.

**No**.	**Study**	**Country**	**N (IG; CG)**	**Age (IG; CG)**	**Intervention (IG)**			**Intervention (CG)**			**Population**	**Outcomes**
					**Intervention content**	**Intervention time, frequency, period**	**Type**	**Intervention content**	**Intervention time, frequency, period**	**Type**		
1	Ahu et al., 2024	Turkey	18;18	51.39 ± 3.65; 52.72 ± 4.39	Laugher yoga	4 weeks, twice a week, 40 to 45 min each time	Mind body exercise	NI	4 weeks	NI	Menopausal Women	PSQI
2	Pauline et al., 2023	Switzerland	15;15	46.6 ± 5.67; 44.8 ± 7.82	Walking	12 weeks, three times a week, 75 min each time	Aerobic exercise	NI	12 weeks	NI	Adult women	ISI
3	María et al., 2022	Spain	57;60	69.70 ± 6.15; 69.75 ± 6.7	Qigong	12 weeks, twice a week, 60 min each time	Mind body exercise	NI	12 weeks	NI	Postmenopausal women	PSQI
4	Rui et al., 2012	Brazil	14;15	50–65; 50–65	Stretching	16 weeks, twice a week, 60 min each time	Stretching exercise	NI	16 weeks	NI	Postmenopausal women	ISI
4	Rui et al., 2012	Brazil	15;15	50–65; 50–65	Yoga	16 weeks, twice a week, 60 min each time	Mind body exercise	NI	16 weeks	NI	Postmenopausal women	ISI
5	Steriani et al., 2007	USA	55;54	50.5 ± 3.6; 48.6 ± 3.6	Walking	16 weeks, three times a week, 60 min each time	Aerobic exercise	NI	16 weeks	NI	Middle-aged women	PSQI
5	Steriani et al., 2007	USA	55;54	50.5 ± 3.6; 48.6 ± 3.6	Yoga	16 weeks, twice a week, 90 min each time	Mind body exercise	NI	16 weeks	NI	Middle-aged women	PSQI
6	Barbara et al., 2014	USA	103;138	55.8 (3.6); 54.2 (3.5)	Running and cycling	12 weeks, three times a week, 75 min each time	Aerobic exercise	NI	12 weeks	NI	Menopausal Women	PSQI
7	Azam et al., 2023	Iran	32;35	20.75 ± 1.27; 20.08 ± 1.31	Pilates	8 weeks, once a week, 60 min each time	Mind body exercise	NI	8 weeks	NI	Female students	PSQI
8	Curi et al., 2018	Brazil	31;30	64.25± 0.14; 63.75 ± 0.08	Pilates	16 weeks, twice a week, 60 minutes each time	Mind body exercise	NI	16 weeks	NI	Elderly women	PSQI
9	Maryam et al., 2020	Iran	32;35	20.53 ± 1.60; 20.08 ± 1.31	Different aerobic exercises	8 weeks, 3 times a week, 60 minutes each time	Multi mode motion	NI	8 weeks	NI	Female student	PSQI
10	Tadayon et al., 2016	Iran	56;56	52.3 ± 1.6; 52.48 ± 1.7	Walking	12 weeks, increase your steps by 500 each week. By the end of 12 weeks, you can take up to 10,000 steps per week at mos	Aerobic exercise	NI	12 weeks	NI	Menopausal women	PSQI
11	Jae et al., 2019	Korea	10;10	64.10 ± 3.35; 63.20 ± 2.62	Resistance training and cycling	12 weeks, three times a week, 60 min each time	Multi mode motion	NI	12 weeks	NI	Old women	PSQI
11	Jae et al., 2019	Korea	10;10	65.20 ± 5.10; 63.20 ± 2.62	Resistance training and cycling	12 weeks, three times a week, 60 min each time	Multi mode motion	NI	12 weeks	NI	Old women	PSQI
12	Shu et al., 2012	Taiwan	35;35	Over 45 years old; Over 45 years old	Qigong	12 weeks, 7 times a week, 30 min each time	Mind body exercise	NI	12 weeks	NI	Perimenopausal Women	PSQI
13	Jiaojiao et al., 2017	China	23;23	64.61(3.40); 64.53(3.43)	Tai Chi	24 weeks, three times a week, 60 min each time	Mind body exercise	NI	24 weeks	NI	Elderly women	PSQI
14	Agustín et al., 2019	Spain	55;52	69.98 ± 7.83; 66.79 ± 10.14	Pilates	12 weeks, twice a week, 60 min each time	Mind body exercise	NI	12 weeks	NI	Postmenopausal women	PSQI
15	Shelly et al., 2024	India	25;25	45–60; 45–60	Walking	6 weeks, 3 times a week, 30 min each time	Aerobic exercise	NI	6 weeks	NI	Postmenopausal Women	PSQI

IG, intervention group; CG, control group; N, Number; NI, No Intervention; PSQI, Pittsburgh sleep quality index; ISI, Insomnia Severity Index.

In order to investigate whether different types of physical activity exert different impacts on women's sleep quality, they were classified into 4 categories based on the common characteristics of physical activity and prior relevant studies (De Zoete et al., 2021; Nie et al., 2024). The classification in this study mainly involves aerobic exercise, which is defined as exercise that primarily relies on aerobic metabolism to supply the energy needed for physical activity (Baron et al., 2023; Elavsky and McAuley, 2007; Sharma et al., 2024; Sternfeld et al., 2014; Tadayon et al., 2016). Multi mode motion refers to intervention that involves the use of two or more types of exercises, such as combinations of aerobic exercise and resistance exercise (Ezati et al., 2020; Yoon et al., 2019). Stretching exercises help maintain the flexibility and extensibility of joints, and increase joint range of motion, reducing the risk of injury (Afonso et al., 2012). Mind body exercise is a movement practice focused on deepening one's own body awareness, including techniques such as body awareness training and breathing exercises (Elavsky and McAuley, 2007; Afonso et al., 2012; Aibar-Almazán et al., 2019; Aksoy-Can et al., 2025; Amzajerdi et al., 2023; Curi et al., 2018; Carcelén-Fraile et al., 2022; Lü et al., 2017; Yeh and Chang, 2012).

### 3.3 Risk of bias

**Figure 2:**
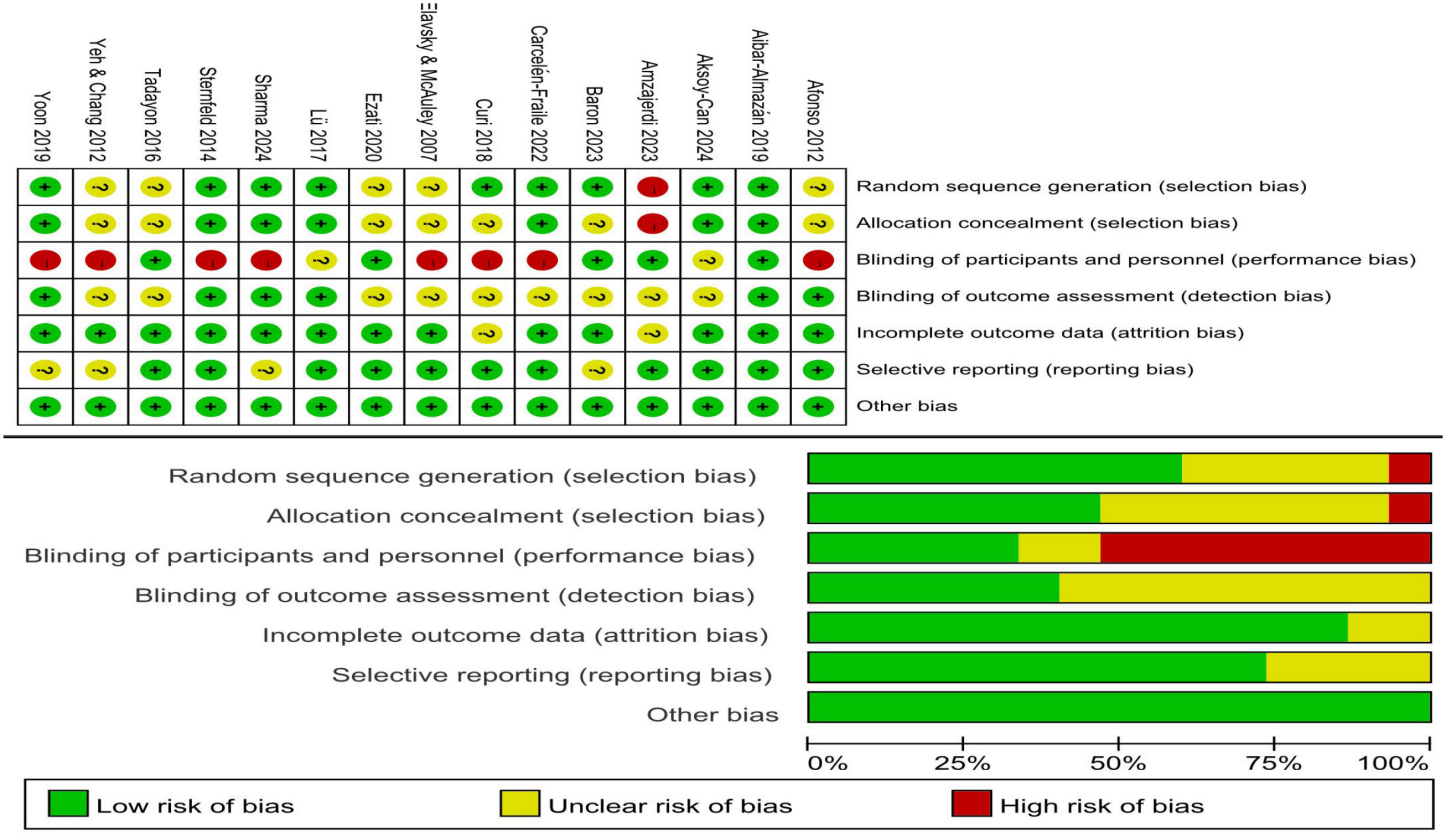
Risk of bias of included studies.

Among the 15 studies, for random sequence generation, approximately 9 studies were deemed to have a low risk of bias, 5 had an unclear risk, and 1 had a high risk. For allocation concealment, around 7 studies were considered to have a low risk of bias, 7 had an unclear risk, and 1 had a high risk. Regarding blinding of participants and personnel, 5 studies were judged to have a low risk of bias, 2 had an unclear risk, and 8 had a high risk. For blinding of outcome assessment, 6 studies were considered to have a low risk of bias, and 9 had an unclear risk. For incomplete outcome data, 14 studies were deemed to have a low risk of bias, and 1 had an unclear risk. For selective reporting, 11 studies were considered to have a low risk of bias, and 4 had an unclear risk. All 15 studies were classified as having a low risk of other bias. The specific bias assessment results are presented in Figure 2, which detail the scores and classifications of each study according to each bias risk criterion.

### 3.4 Network meta-analysis

**Figure 3:**
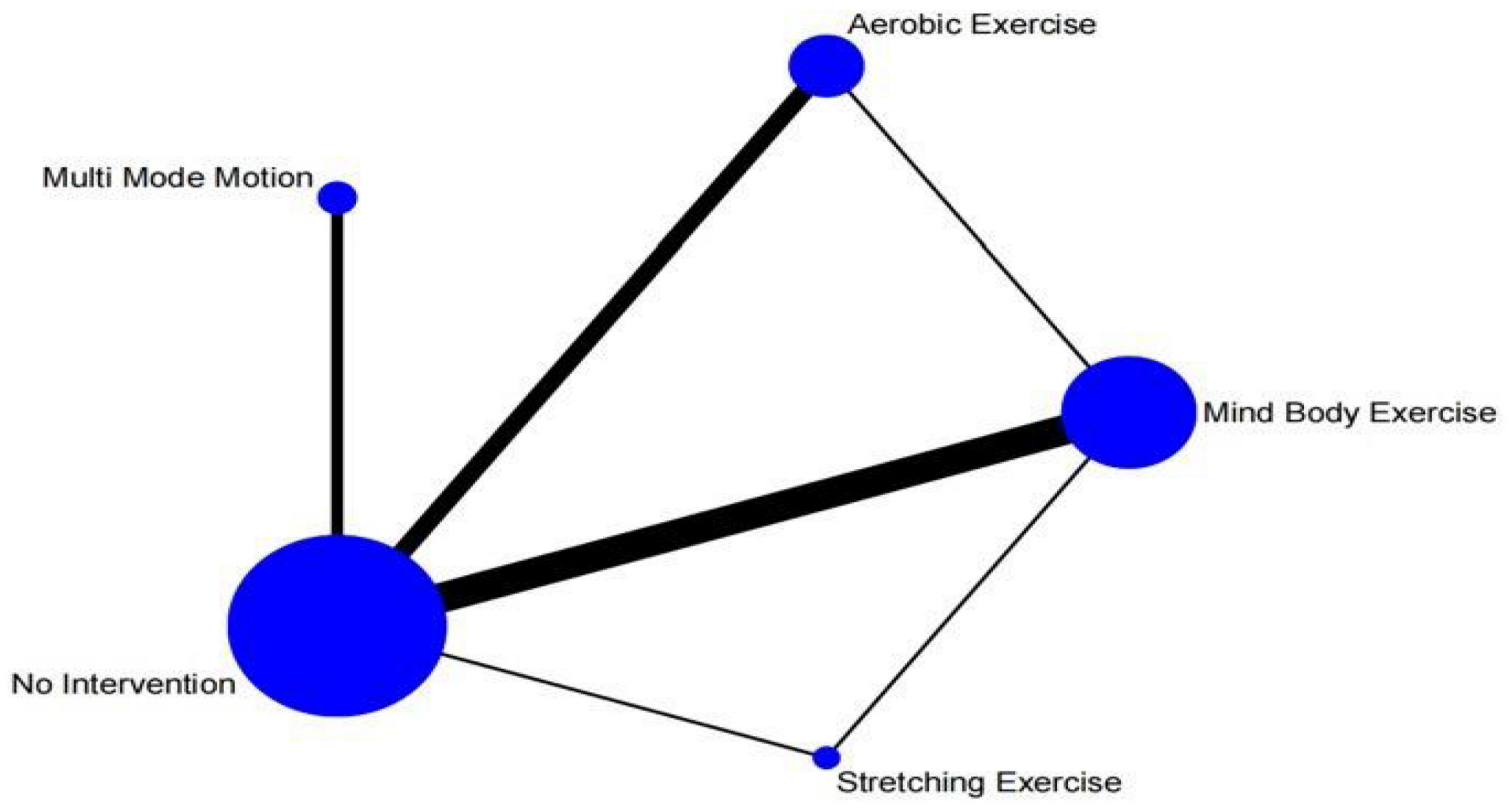
Network diagram.

Figure 3 The network meta-analysis graph is presented. Apparently, among the experimental group, the three intervention measures with the largest sample sizes are aerobic exercise, multi mode motion, and Mind body exercise. In the control group, the intervention with the largest sample size is no intervention, which means maintaining daily activities. The most frequently studied comparisons are between aerobic exercise and maintaining daily activities, as well as between Mind body exercise and maintaining daily activities.

**Figure 4:**
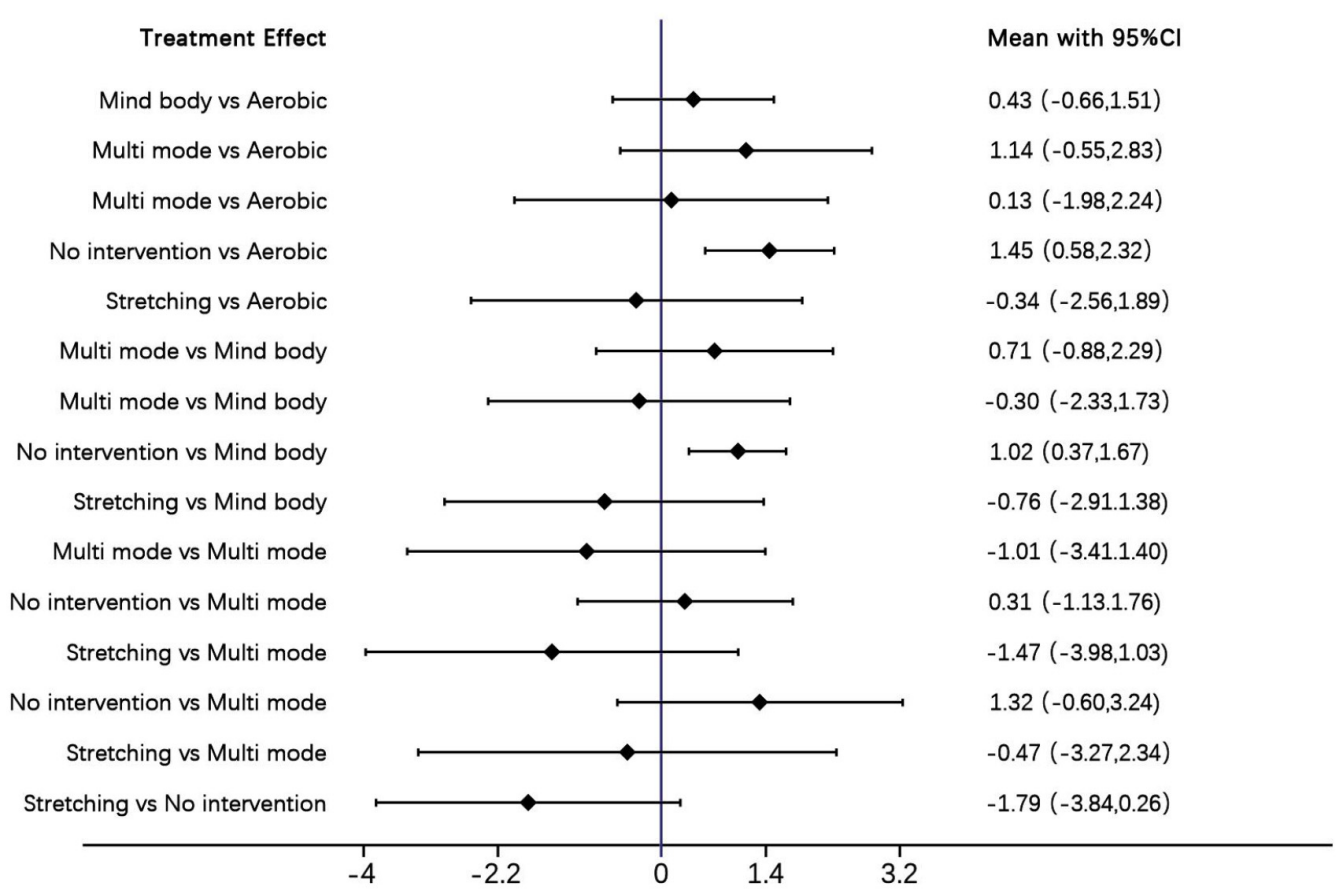
Forest plot.

The forest plot compares the SMD with 95% confidence intervals (95% CIs) of physical activity interventions for sleep quality in women, and presents direct and indirect analyses (Figure 4). Aerobic exercise and multi mode motion are more effective than control conditions; a higher SMD value indicates a better therapeutic effect.

The following results report the effect size magnitudes (SMD with 95% CIs) for comparisons between different exercise interventions. Aerobic exercise showed greater improvement compared to no intervention (SMD = 3.39 [95% CI: 1.08, 5.81]); this effect translated to clinically meaningful sleep improvement, as it can help most women with poor sleep quality (per PSQI criteria) transition to good sleep and alleviate insomnia related symptoms significantly and stretching exercise (SMD = 1.81 [95% CI: −3.38, 7.13]), as well as an advantage over Mind body exercise (SMD = 0.84 [95% CI: −1.94, 3.74]) and multi mode motion (SMD = 0.19 [95% CI: −3.90, 4.38]). Mind body exercise is more effective than no intervention (SMD = 2.55 [95% CI: 0.74, 4.37]) it exerts tangible clinical benefits, such as reducing sleep disruptions linked to hormonal fluctuations (e.g., in perimenopausal women) and relieving mild to moderate insomnia symptoms but shows no clear advantage over aerobic exercise (SMD = −0.84 [95% CI: −3.74, 1.94]), multi–mode motion (SMD = −0.65 [95% CI: −4.48, 3.22]), or stretching exercise (SMD = 0.97 [95% CI: −3.78, 5.72]). Multi mode motion demonstrates improvement compared to no intervention (SMD = 3.20 [95% CI: −0.20, 6.59]) and stretching exercise (SMD = 1.62 [95% CI: −4.26, 7.45]) its clinical value lies in addressing both physical discomfort (e.g., muscle stiffness) and psychological stress that disrupt sleep, making it suitable for subgroups like elderly women but does not significantly differ from aerobic exercise (SMD = −0.19 [95% CI: −4.38, 3.90]) or Mind body exercise (SMD = 0.65 [95% CI: −3.22, 4.48]). No intervention is consistently less effective, performing worse than aerobic exercise (SMD = −3.39 [95% CI: −5.81, −1.08]) and Mind body exercise (SMD = −2.55 [95% CI: −4.37, −0.74]), and showing no advantage over multi mode motion (SMD = −3.20 [95% CI: −6.59, 0.20]) or stretching exercise (SMD = −1.58 [95% CI: −6.33, 3.16]). Stretching exercise has no significant advantage compared to aerobic (SMD = −1.81 [95% CI: −7.13, 3.38]), mind–body (SMD = −0.97 [95% CI: −5.72, 3.78]), or multi mode motion (SMD = −1.62 [95% CI: −7.45, 4.26]), but is superior to no intervention (SMD = 1.58 [95% CI: −3.16, 6.33]) its clinical effect is limited, though, as it only slightly improves sleep initiation and has little impact on key sleep indicators like sleep duration and efficiency (Table 2).

**Table 2:** League table on interventions.

**Aerobic exercise**	**Multi mode motion**	**Mind body exercise**	**Stretching exercise**	**No intervention**
Aerobic exercise	0.19 (−3.9, 4.38)	0.84 (−1.94, 3.74)	1.81 (−3.38, 7.13)	3.39 (1.08, 5.81)
−0.19 (−4.38, 3.9)	Multi mode motion	0.65 (−3.22, 4.48)	0.97 (−3.78, 5.72)	2.55 (0.74, 4.37)
−0.84 (−3.74, 1.94)	−0.65 (−4.48, 3.22)	Mind body exercise	1.62 (−4.26, 7.45)	3.2 (−0.2, 6.59)
−1.81 (−7.13, 3.38)	−1.62 (−7.45, 4.26)	−0.97 (−5.72, 3.78)	Stretching exercise	1.58 (−3.16, 6.33)
−3.39 (−5.81, −1.08)	−3.2 (−6.59, 0.2)	−2.55 (−4.37, −0.74)	−1.58 (−6.33, 3.16)	No intervention

**Figure 5:**
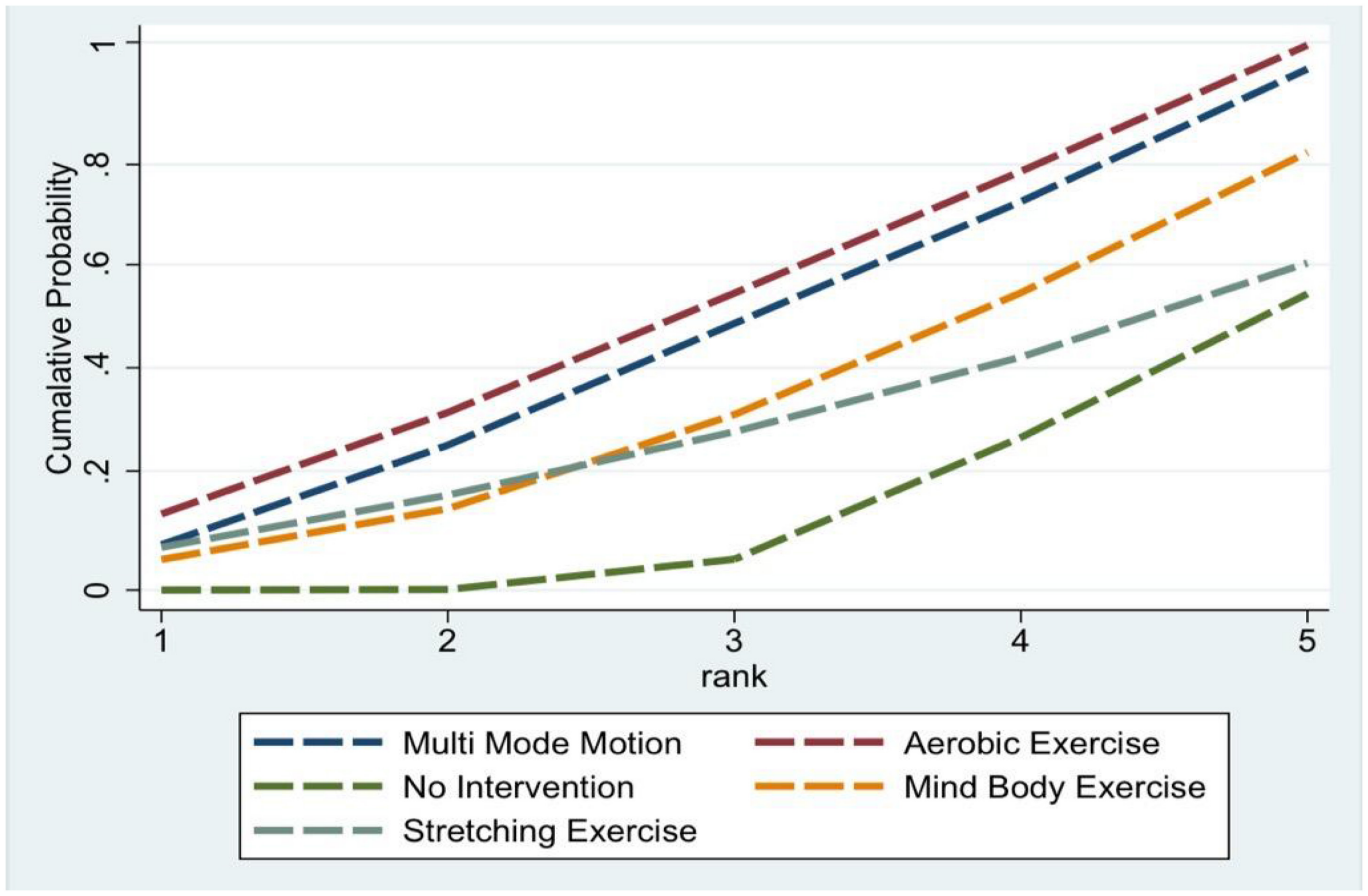
SUCRA plot.

These SMD values quantify differences in sleep quality among women across interventions, where larger absolute values reflect stronger effects. Regarding the probability of different interventions improving sleep, SUCRA rankings further clarified the likelihood of each intervention being the most effective. Aerobic exercise ranked first (SUCRA = 77.2%), followed closely by multi mode motion (SUCRA = 70.5%). Mind body exercise and stretching exercise rank third and fourth, respectively (SUCRA = 57.1% and 38.8%). The probability of being the “best treatment” is highest for aerobic exercise (40.9%), multi mode motion (37.4%), and stretching exercise (13.0%). Together, these three account for 91.3%, with specific results shown in Figure 5.

### 3.5 Publication bias

**Figure 6:**
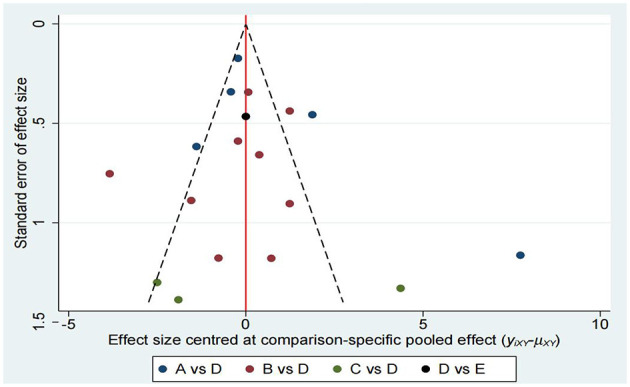
Funnel plot on publication bias.

As shown in Figure 6, a funnel plot was first used to assess publication bias. The studies in the funnel plot were roughly symmetrically distributed, and no obvious signs of publication bias were found through visual inspection. This suggests that although there might be some publication bias in the original data, its impact is not significant. Overall, the estimated effect size is still statistically significant, indicating that the study results are somewhat robust.

Subsequently, we conducted Begg's test and Egger's test to assess potential bias in the study. Begg's test showed no significant bias, while Egger's test indicated significant bias (see Appendix C1). These tests were selected because they address publication bias through different statistical methods: Begg's test (rank-based) is less sensitive to small-sample heterogeneity, whereas Egger's test (regression-based) may be more effective at detecting bias in larger datasets. Their conflicting results may reflect differences in statistical power and methodological assumptions.

To more comprehensively assess publication bias, we conducted a trim-and-fill analysis using a random-effects model. The results of the study showed that after adjusting for publication bias via the trim-and-fill method, the estimated effect size decreased slightly, though the change was minimal. This method supplements funnel plots and statistical tests by empirically estimating hypothetical missing studies, thereby addressing the limitations of purely visual or parametric approaches. The estimated effect size remained statistically significant, which further supports the robustness of the study results, as presented in Appendices C2 and C3.

## 4 Discussion

This network meta-analysis comprehensively evaluated the effects of various physical activity interventions on women's sleep. Based on a comprehensive analysis of 15 RCTs, our findings indicate that aerobic exercise, multi mode motion, and Mind body exercise are the most effective non-pharmacological interventions for improving women's sleep. Notably, aerobic exercise is the intervention most likely to achieve optimal outcomes (SUCRA = 77.2%), followed closely by multi mode motion (SUCRA = 70.5), which reflects its synergistic potential in improving women's sleep. Mind body exercise ranks third (SUCRA = 57.1%) (Table 3).

**Table 3:** Ranking of SUCRA probabilities.

**Intervention name**	**SUCRA (%)**	**PrBest (%)**	**Mean rank**
Aerobic exercise	77.2	40.9	1.9
Multi mode motion	70.5	37.4	2.2
Mind body exercise	57.1	8.7	2.7
Stretching exercise	38.8	13.0	3.4
No intervention	6.4	0.0	4.7

Sleep quality and physical activity are crucial for women to maintain their health (Liu, 2016; Consensus Conference Panel et al., 2015). For ordinary women, the impacts of different physical activity interventions on improving sleep quality still require comprehensive evaluation. In particular, there is no relevant network meta-analysis in the current existing studies. This paper aims to eliminate the impact of objective factors such as other diseases, provide the optimal exercise intervention measures to improve the sleep quality of the general female population, and offer exercise prescription recommendations for improving sleep quality to a broader population.

Studies have shown that aerobic exercise interventions can significantly improve women's sleep quality (Baron et al., 2023; Elavsky and McAuley, 2007; Sharma et al., 2024; Sternfeld et al., 2014; Tadayon et al., 2016). The core mechanisms behind this are regulating the body temperature rhythm, reducing bodily inflammation, and balancing stress hormones (Baron et al., 2023). During aerobic exercise, body temperature rises more rapidly than in other forms of physical activity (Sternfeld et al., 2014). Subsequently, it gradually returns to the normal core body temperature within a few hours after exercise (Elavsky and McAuley, 2007). This fluctuation in body temperature mimics the human body's natural circadian rhythm and can extend the duration of deep sleep in women (Reid et al., 2021). In addition, regular aerobic exercise can be used to control systemic inflammation, thereby reducing the negative impact of inflammation on sleep (Mathur and Pedersen, 2008). Furthermore, aerobic exercise can also inhibit the excessive secretion of stress hormones (cortisol) and enhance the activity of the brain's “relaxation factors” (e.g., serotonin), which can effectively alleviate difficulty falling asleep caused by anxiety (De Nys et al., 2022). Aerobic exercise not only strengthens women's physical fitness but also effectively relieves their psychological anxiety (Bartlewski, 1996), which helps to improve their sleep quality.

Multi mode motion is a composite intervention method that includes two or more types of exercise (Solis-Navarro et al., 2023). It has significant value in physical activity interventions for improving women's sleep quality, ranking second in terms of sleep improvement effect—only inferior to aerobic exercise but significantly better than Mind body exercise and stretching exercise, which further confirms its advantages. It has an extremely wide population adaptability: for elderly women, the combination of “resistance training such as sit-ups and squats + cycling interval training” is used to effectively improve sleep quality (Yoon et al., 2019); for female college students, the combined program of “10-min warm-up + 35-min basic aerobic exercise + 15-min relaxation” also achieves positive effects (Ezati et al., 2020). multi mode motion addresses the issue of insufficient adaptability of a single type of exercise to various populations (Baker et al., 2007). At the mechanism level, it achieves synergy among different types of exercise: it not only inherits the effects of aerobic exercise in regulating body temperature rhythm and reducing systemic inflammation to extend deep sleep duration (Korkutata et al., 2025), but also enhances muscle strength through resistance training and alleviates physical discomforts such as low back pain to reduce sleep interruptions (Vogel et al., 2021). Psychologically, it reduces monotony and improves compliance by switching exercise types, and can also balance cognitive load, avoid psychological stress caused by high-intensity single exercise, and indirectly reduce difficulty falling asleep due to anxiety (Kröz et al., 2017). In terms of application, it has extremely high flexibility: its intensity can be adjusted according to exercise capacity (e.g., elderly women use bodyweight resistance training instead of weighted resistance training), it can be combined with stretching exercise to enhance the effect, and it requires no special equipment and can be carried out in communities or families (Buman and King, 2010).

Mind body exercise also has a positive impact on women's sleep quality (Wang et al., 2020; Yeh and Chang, 2012; Buchanan et al., 2017; Jain and Sundarajan, 2022; James et al., 2022; Leung et al., 2021; Jorge et al., 2016; Streeter et al., 2007; Wong et al., 2018). In particular, it can exhibit intervention effects during the sensitive phases of the female neuroendocrine system (Jorge et al., 2016). At the neuroendocrine level, Mind body exercise can reduce the circadian secretion of the stress hormone cortisol by inhibiting the excessive activation of the hypothalamus, pituitary gland, and adrenal gland (Streeter et al., 2007). Meanwhile, it can improve the synthesis efficiency of gamma-aminobutyric acid (GABA), an inhibitory neurotransmitter in the central nervous system, thereby alleviating psychological emotions such as anxiety and indirectly reducing sleep-disturbing factors (Wong et al., 2018). At the level of autonomic nervous regulation, the combination of abdominal breathing and low-intensity Mind body exercise can enhance the regulatory capacity of the vagus nerve on the heart, promote the transition of the autonomic nervous system from an excited state, and effectively improve sleep quality (Afonso et al., 2012).

Studies have shown that stretching exercise has a weak sleep-promoting mechanism (Mohammad et al., 2024), and its core functions are only defined as “maintaining joint flexibility and extensibility, increasing joint range of motion, and reducing the risk of sports injuries” (Page, 2012). It has not formed regulatory mechanisms for core sleep problems in women, such as “sleep rhythm disorders, difficulty falling asleep caused by anxiety, and sleep interruptions caused by physical discomfort” (Rady et al., 2020); it has neither rhythm regulation nor hormonal balance effects at the physiological level, nor emotional relief ability at the psychological level, and can only indirectly reduce a small amount of physical disturbances by improving muscle stiffness (Driver and Taylor, 2000). Furthermore, its improvement magnitude on women's sleep quality is relatively slight (Komatsuzaki et al., 2024), and there are a large number of more effective alternative options in the non-pharmacological intervention system: those seeking effective sleep improvement can choose aerobic exercise (Elavsky and McAuley, 2007; Sharma et al., 2024), while those with weaker exercise capacity or who need to take into account multiple health needs can choose multi mode motion or Mind body exercise. These alternative options not only have stronger sleep improvement effects, but also additionally bring overall health benefits such as “improving cardiopulmonary function, enhancing muscle strength, and alleviating psychological anxiety” (Baron et al., 2023; Solis-Navarro et al., 2023; Jorge et al., 2016), whereas stretching exercise has no significant sleep-promoting advantages compared with other types of exercise. In addition, from the perspective of the “cost-effectiveness ratio” in community health management, the input and output of promoting stretching exercise are not proportional (Roine et al., 2009; Wang et al., 2025); under the same guidance and time costs, aerobic exercise or multi mode motion can help more women achieve more significant sleep improvement. Therefore, stretching exercise lacks practical value in clinical recommendation and community promotion, and it is difficult to become a priority intervention method for improving women's sleep.

To summarize, the key findings and comprehensive practical implications of this study focused on general adult women (excluding those with chronic diseases or severe sleep disorders) are as follows: Based on the analysis of 15 high-quality randomized controlled trials (RCTs), aerobic exercise is the most effective non-pharmacological intervention for improving women's sleep quality (SUCRA = 77.2%), closely followed by multi mode motion (SUCRA = 70.5%). Mind body exercise exhibits a moderate improvement effect (SUCRA = 57.1%), while stretching exercise is the least effective (SUCRA = 38.8%). This result not only clarifies the priority ranking of different exercise interventions but also holds three core values in practical scenarios: In clinical practice, it provides clinicians with a clear non-pharmacological intervention plan. There is no need to rely entirely on sedative medications (which may lead to dependence); instead, aerobic exercise (e.g., brisk walking, cycling) can be prioritized for most women. For elderly women or those with limited exercise capacity, multi mode motion (such as “resistance training + aerobic exercise”) is recommended, as it is safe and can address sleep issues in a targeted manner. In community health management, the high feasibility of these interventions can be directly translated into actionable measures: Aerobic exercises and multi mode motion require no special equipment, so communities can promote them by organizing group exercise classes, distributing simple exercise guides, and other means. This helps a large number of women improve sleep in their daily lives and reduces subsequent health risks caused by poor sleep (e.g., anxiety, metabolic abnormalities). For ordinary women, the study results eliminate confusion about “which type of exercise to choose for sleep improvement” there is no need to try multiple methods blindly. They can start with aerobic exercise, and if they prefer low-intensity activities, mind body exercise is an alternative. This makes sleep improvement more targeted and operable. These findings fill the gap in existing research, which lacks precise guidance for “sleep interventions in general women.” They transform abstract research data into actionable strategies applicable in clinical settings, communities, and individual daily lives, providing a reference that is both scientific and practical for improving women's sleep quality.

## 5 Strengths and limitations

This study has several strengths: it is the first network meta-analysis exploring the effects of physical activity interventions on women's sleep quality, combines multiple studies to enhance result reliability, and focuses on RCTs to strengthen result stability. However, limitations exist: while funnel plots showed roughly symmetric distribution and trim-and-fill analysis indicated minimal effect size changes after adjustment, Egger's test (not Begg's) detected publication bias likely from unreported small-sample studies that slightly weakens conclusion robustness; despite using an inverse variance weighted random effects model to address scale heterogeneity, unstandardized intervention protocols and unstratified participant subgroups have caused residual heterogeneity, reducing inter-intervention comparability; additionally, only English full-text studies were included, excluding potential high quality non-English RCTs and restricting generalizability to non-English-speaking populations; the total sample size of included studies is also small, weakening statistical power for subgroup analyses.

Future research can focus on several important areas. First, personalized physical activity intervention measures tailored to the different characteristics of specific female groups may be more effective. For example, when selecting appropriate intervention measures, healthcare providers should consider factors such as the patient's physical condition, age, and the severity of the illness. physical activity programs customized according to these individual variables can improve sleep quality. Second, researchers should conduct further research on the optimal parameters of physical activity, such as frequency, duration, and intensity, to explore the optimal dosage of intervention measures.

## 6 Conclusion

This study confirms that aerobic exercise is the most effective non-pharmacological intervention measure to improve women's sleep quality, with Mind body exercise coming in second. In clinical practice, aerobic exercise is recommended as the top choice. Alternatively, Mind body exercise or multi mode motion can be selected based on patients' individual characteristics (such as insomnia and exercise capacity). Meanwhile, intervention parameters (such as frequency and intensity) should be standardized to enhance reproducibility. In residential communities, the significance of aerobic exercise for women's sleep can be promoted through publicity, offering suggestions to improve the sleep quality of a large number of women. Future research should focus on developing personalized exercise programs (combining genes, phenotype, and cognitive baseline), observing the long-term therapeutic effect, and researching the Intervention Mechanism to further optimize strategies for improving women's sleep quality.

## Data Availability

The original contributions presented in the study are included in the article/Supplementary material, further inquiries can be directed to the corresponding authors.
